# Differential Impact of Constrictive Physiology after Pericardiocentesis in Malignancy Patients with Pericardial Effusion

**DOI:** 10.1371/journal.pone.0145461

**Published:** 2015-12-21

**Authors:** In-Jeong Cho, Hyuk-Jae Chang, Hyemoon Chung, Sang-Eun Lee, Chi Young Shim, Geu-Ru Hong, Jong-Won Ha, Namsik Chung

**Affiliations:** 1 Department of Internal Medicine, Division of Cardiology, Severance Hospital, Yonsei University College of Medicine, Seoul, Republic of Korea; 2 Severance Biomedical Science Institute, Yonsei University College of Medicine, Seoul, Republic of Korea; Peking Union Medical College Hospital, CHINA

## Abstract

**Background:**

Echocardiographic signs of constrictive physiology (CP) after pericardiocentesis are frequently observed in malignancy patients. The purpose of the current study was to explore whether features of CP after pericardiocentesis have prognostic impact in malignancy patients with pericardial effusion (PE).

**Methods:**

We retrospectively reviewed 467 consecutive patients who underwent pericardiocentesis at our institution from January 2006 to May 2014. Among them, 205 patients with advanced malignancy who underwent comprehensive echocardiography after the procedure comprised the study population. Co-primary end points were all-cause mortality (ACM) and repeated drainage (RD) for PE. Patients were divided into four subgroups according to cytologic result for malignant cells and CP (positive cytology with negative CP, both positive, both negative, and negative cytology with positive CP).

**Results:**

CP after pericardiocentesis was present in 106 patients (50%) at median 4 days after the procedure. During median follow-up of 208 days, ACM and RD occurred in 162 patients (79%) and 29 patients (14%), respectively. Cox regression analysis revealed that independent predictors for ACM were male gender and positive cytology (all, p < 0.05). For RD, predictors were positive cytology, the absence of cardiac tamponade, and negative CP after pericardiocentesis (all, p < 0.05). When the patients were divided into four subgroups, patients with negative cytology and positive CP demonstrated the most favorable survival (hazard ratio [HR]: 0.39, p = 0.005) and the lowest RD rates (HR: 0.07, p = 0.012).

**Conclusion:**

CP after pericardiocentesis is common, but does not always imply poor survival or the need for RD in patients with advanced malignancies. On the contrary, the presence of CP in patients with negative cytology conferred the most favorable survival and the lowest rate of RD. Comprehensive echocardiographic evaluation for CP after pericardiocentesis would be helpful for predicting prognosis in patients with advanced malignancies.

## Introduction

Cardiac tamponade is an acute or subacute compression of the heart due to pericardial fluid accumulation and can be life-threatening. The most common cause of cardiac tamponade is malignancy, which is involved in > 50% of all tamponade cases[1]. Pericardiocentesis is lifesaving in cases of cardiac tamponade; it alone frequently results in the resolution of large pericardial effusion (PE) in malignancy patients, but recurrence is common[2–7].

Constrictive physiology (CP) is an echocardiographic criterion for the diagnosis of constrictive pericarditis and comprehensive transthoracic echocardiography has contributed to the ability of physicians to easily detect constrictive pericarditis without invasive catheterization or operative findings. Classic constrictive pericarditis is known to be a progressive and debilitating condition and is characterized by pericardial fibrosis and calcification[8]. Meanwhile, echocardiographic CP is not only found in patients with symptomatic constrictive pericarditis, but is also more commonly observed in rather benign conditions, especially in post-cardiac surgery patients without clinical constrictive pericarditis when assessed with comprehensive examination[9]. Postoperative CP after cardiac surgery has been reported to be relatively common and is usually transient and benign[9].

The predictors of mortality and recurrence of PE following pericardiocentesis in malignancy patients are not yet fully understood. Survival of patients with malignancy and PE has been reported to be mainly associated with the extent of the disease, reflected by positive results on PE cytology[10]. Although CP is also frequently observed in malignancy patients after pericardiocentesis, few data are available regarding the clinical implications of echocardiographically observed CP after pericardiocentesis. Therefore, the purpose of the current study was to investigate whether features of CP after pericardiocentesis have prognostic implications for all-cause mortality (ACM) and repeated drainage (RD) of PE after pericardiocentesis in malignancy patients. We hypothesized that the prognosis of malignancy patients with large PE would be different according to the presence of CP and cytologic results for malignant cells after pericardiocentesis.

## Methods

### Patient population

We retrospectively reviewed data from 467 patients who underwent pericardiocentesis due to PE at our institution from January 2006 to May 2014. After excluding 232 patients without malignancies, 12 patients without postoperative comprehensive echocardiography, 9 patients with acute leukemia, 4 patients with lymphoma and 5 patients with stage I, II or complete remission status, the remaining 205 patients with advanced solid malignancies who underwent comprehensive echocardiography after the pericardiocentesis comprised the study population. Charts were systemically reviewed for data relating to cancer stage, co-morbidities, and laboratory studies such as hemoglobin, creatinine, and C-reactive protein (CRP). Cardiac tamponade was defined by combining clinical and echocardiographic criteria[11]. Characteristic clinical criteria included venous hypotension, pulsus paradoxus, or arterial hypotension in the absence of other possible causative conditions and resolution of the syndrome on removal of PE. Along with the characteristic clinical criteria, echocardiographic criteria, such as chamber collapse, mitral inflow respiratory variation, and inferior vena cava plethora, were also assessed to diagnose cardiac tamponade [12].

Co-primary end points were ACM and RD. All PE was confirmed by pre-procedural echocardiography. Pericardiocentesis was performed under echocardiographic or fluoroscopic guidance. Prolonged PE drainage was done for all patients as follows: The pericardial catheter was left in place until complete drainage of PE was obtained, which was defined as drainage of ≤ 40 ml over a 24-hour period and a follow-up 2-dimentional echocardiogram with residual pericardial effusion, which was non-circumferential and < 1 cm in size. Values of carcinoembryonic antigen (CEA) and cytokeratin fragment 19 (CYFRA 21–1) were measured in drained pericardial effusion. None of the patients were treated by instillation of sclerosing agent following pericardiocentesis. Patients were divided into four subgroups according to cytologic result for malignant cells and echocardiographic CP after pericardiocentesis (positive cytology with negative CP, both positive, both negative, and negative cytology with positive CP). Patient records/information was anonymized and de-identified prior to analysis. This study was approved by the Institutional Review Board of Yonsei University, Severance Hospital, Seoul, Korea.

### Echocardiographic measurement

Standard 2-dimensional and Doppler echocardiographic assessments were performed. The echocardiographic images of the included patients were reanalyzed by 2 experienced echocardiographers who were blinded to patient clinical data. Left ventricular (LV) internal diameter, septal thickness, and LV posterior wall thickness were measured at end-diastole. LV mass was calculated as recommended by the American Society of Echocardiography[13], and LV mass was indexed for body surface area. The left atrial volume was calculated from the parasternal long-axis view and apical four-chamber view using the prolate ellipse method[14] and was indexed for the body surface area. The mitral inflow velocities were obtained by pulsed wave Doppler in the apical four-chamber view. The mitral early diastolic velocity (E) was measured. The peak early diastolic mitral annular (E') velocity was measured from the septal mitral annulus; we then calculated the E/E' ratio. A moderate pericardial effusion was considered to be present when the sum of the diastolic echo-free anterior and posterior spaces was 1 to 2 cm and large when it was greater than 2 cm[12]. CP was defined as follows: respiratory-related ventricular septal shift, dilated inferior vena cava (maximal diameter > 21 mm), a respiratory variation of 25% or more in the mitral inflow E velocity and increased diastolic flow reversal with expiration in the hepatic vein, and mitral septal annulus velocity of more than 7 cm/sec [15,16].

### Pericardial wall thickening and tumor infiltration

Maximal pericardial thickness was measured using computed tomography (CT) with contrast that enabled assessment of the whole pericardium. Pericardial thickening was defined as a pericardial thickness of more than 4 mm [11,17]. Irregular nodular thickening or tumor infiltration of the pericardium was assessed using CT findings to determine whether there was metastatic involvement of the pericardium [18].

### Statistical analysis

Distribution of relevant variables was reported either as a percentage or as the mean ± standard deviation. The groups were compared using χ^2^ statistics for categorical variables and the Student t test for continuous variables. Multivariable Cox proportional hazard analysis was employed to determine independent variables for ACM and RD, with variables with P < 0.3 in univariate analysis between the patients with and without primary outcomes as covariates. For Kaplan-Meier analysis, we analyzed all combined events, ACM and RD by time to the first event. P-values < 0.05 were considered statistically significant.

## Results

### Patient characteristics

The indications for drainage were cardiac tamponade in 167 patients (81%), relief of an impending cardiac tamponade in 33 patients (16%), and diagnostic purposes in patients without a clear etiology in five patients (2%). Patient characteristics and comparison of characteristics in patient groups according to all combined events, ACM and RD are shown in Table 1. Of the 205 patients included in this study, there were 121 men and 84 women with a mean age of 58 ± 13 years. CP after pericardiocentesis was present in 102 patients (50%) at median 4 days (1–27 days) after the procedure. During a median follow-up of 208 days (1–2408 days), ACM and RD occurred in 162 patients (79%) and 29 patients (14%), respectively. Indication for RD was cardiac tamponade in all the 29 patients with RD.

**Table 1:** Comparison of characteristics in the patient groups according to all combined events, all-cause mortality and repeated drainage of pericardial effusion.

Variable	All combined events			All-cause mortality			Repeated drainage		
	Without events (n = 39)	With events (n = 166)	P-value	Survivor (n = 43)	Non-survivor (n = 162)	P-value	No repeated drainage (n = 176)	Repeated drainage (n = 29)	P-value
Age, years	57 ± 14	58 ± 13	0.857	58 ± 13	58 ± 13	0.936	58 ± 13	56 ± 14	0.337
Male, n (%)	19 (49)	102 (61)	0.146	21 (49)	100 (62)	0.127	108 (66)	13 (45)	0.093
Types of cancer, n (%)			0.821			0.803			0.595
Lung cancer	22 (56)	93 (56)		26 (60)	89 (55)		98 (56)	17 (59)	
Breast cancer	5 (13)	19 (11)		5 (12)	19 (12)		20 (11)	4 (14)	
Stomach cancer	3 (7)	18 (11)		3 (7)	18 (11)		17 (10)	4 (14)	
Esophageal cancer	2 (5)	4 (2)		2 (5)	4 (2)		4 (2)	2 (7)	
Colon cancer	1 (3)	5 (3)		1 (2)	5 (3)		6 (3)	0 (0)	
Cervical cancer	2 (5)	3 (2)		2 (5)	3 (2)		5 (3)	0 (0)	
Other*	4 (10)	24 (14)		4 (9)	24 (15)		26 (15)	2 (7)	
Cancer stage, n (%)			0.977			0.815			0.107
III	5 (13)	21 (13)		5 (12)	21 (13)		25 (14)	1 (3)	
IV	34 (87)	145 (87)		38 (88)	141 (87)		151 (86)	28 (97)	
Hypertension, n (%)	16 (41)	55 (33)	0.351	18 (42)	53 (33)	0.263	62 (35)	9 (31)	0.660
Diabetes mellitus, n (%)	2 (5)	18 (11)	0.279	2 (5)	18 (11)	0.204	19 (11)	1 (3)	0.320
Previous thoracic irradiation, n (%)	12 (31)	40 (24)	0.389	12 (28)	40 (25)	0.667	45 (26)	7 (24)	0.870
Laboratory									
Hemoglobin (g/dL)	11.1 ± 2.1	11.0 ± 1.8	0.814	11.1 ± 2.0	11.0 ± 1.8	0.751	11.0 ± 1.9	11.5 ± 1.5	0.183
Creatinine (g/mL)	0.82 ± 0.40	0.99 ± 1.46	0.465	0.80 ± 0.39	1.00 ±1.47	0.385	1.00 ± 1.42	0.70 ± 0.19	0.257
Cardiac tamponade, n (%)	30 (77)	137 (83)	0.417	31 (72)	136 (84)	0.075	147 (84)	20 (69)	0.062
Pericardial effusion, amount			0.744			0.590			0.324
Moderate	4 (3)	14 (8)		5 (12)	13 (8)		16 (9)	2 (7)	
Large	35 (70)	152 (92)		38 (88)	149 (92)		160 (91)	27 (93)	
Post-pericardiocentesis echocardiographic									
LV end-diastolic diameter (mm)	45.1 ± 5.3	45.0 ± 5.5	0.956	44.9 ± 5.3	45.1 ± 5.5	0.823	45.1 ± 5.2	44.5 ± 6.7	0.608
LV end-systolic diameter (mm)	30.0 ± 5.1	30.6 ± 5.7	0.552	29.8 ± 5.3	30.7 ± 5.7	0.424	30.6 ± 5.2	29.6 ± 7.3	0.392
LV ejection fraction (%)	65.2 ± 9.1	62.5 ± 11.1	0.190	65.0 ± 9.8	62.4 ± 16.4	0.191	62.7 ± 10.4	64.8 ± 12.2	0.357
E velocity (cm/sec)	70.6 ± 16.6	68.7 ± 16.3	0.594	68.9 ± 16.6	69.3 ± 16.4	0.907	69.3 ± 16.6	68.5 ± 15.2	0.852
E' velocity (cm/sec)	7.9 ± 2.6	7.2 ± 2.5	0.249	7.7 ± 2.6	7.3 ± 2.5	0.360	7.6 ± 2.6	6.0 ± 2.0	0.011
E/E'	10.1 ± 4.3	10.9 ± 5.2	0.464	10.0 ± 4.3	11.0 ± 5.2	0.387	10.1 ± 4.1	14.0 ± 7.8	0.002
CP, n (%)	16 (41)	86 (52)	0.226	18 (42)	84 (52)	0.244	94 (53)	8 (28)	0.010
PE fluid cytology, n (%) Positive for malignant cells	19 (49)	117 (70)	0.010	23 (53)	113 (70)	0.045	115 (65)	21 (72)	0.455
Subgroups according to cytology and CP, n (%)			< 0.001			0.001			0.012
Cytology (+), CP (–)	11 (28)	36 (22)		13 (30)	34 (21)		34 (19)	13 (45)	
Cytology (+), CP (+)	7 (18)	81 (49)		9 (21)	79 (49)		80 (45)	8 (28)	
Cytology (–), CP (–)	10 (26)	36 (22)		10 (23)	36 (22)		39 (22)	7 (24)	
Cytology (–), CP (+)	11 (28)	13 (8)		11 (26)	13 (8)		23 (13)	1 (3)	

*Other includes 4 hepatocellular carcinomas, 3 cholangiocarcinoma, 3 renal cell carcinoma, 3 pancreatic cancer, 3 sarcoma, 3 primary unknown cancer, 2 ovarian cancer, 2 parotid gland cancer, 2 malignant thymoma, 1 gallbladder cancer, 1 thyroid cancer, and 1 tonsillar cancer; PE, pericardial effusion; LV, left ventricular; E, early diastolic mitral inflow; E', early diastolic mitral annular; CP, constrictive physiology.

All combined events including ACM and RD occurred in 166 patients (81%). There were no significant differences between the patients with and without all combined events in age, gender, type of cancer, prevalence of hypertension, diabetic mellitus, previous thoracic irradiation, or cardiac tamponade and echocardiographic parameters. Positive cytology was more prevalent in patients with events (p = 0.010). The prevalence of CP was largely comparable between the patients with and without events. When patients were divided into 4 subgroups according to the results of cytology and CP, patients with negative cytology and positive CP demonstrated the lowest all combined events (p < 0.001). Similarly, there were no significant differences in various clinical and echocardiographic characteristics between the survivor and non-survivor groups, except positive cytology (p = 0.045). Patients with negative cytology and positive CP also showed the lowest ACM compared to other groups (p = 0.001).

There were no significant differences between patients with and without RD in age, gender, type of cancer, stage of cancer, or prevalence of hypertension, diabetic mellitus, previous thoracic irradiation and cardiac tamponade. The prevalence of positive cytology was also not significantly different between the two groups, but CP was more frequently observed in patients without RD compared to those with RD (p = 0.010). E' velocity was higher and E/E' was lower in patients without RD (p = 0.002 and p = 0.010, respectively), reflecting exaggerated E' velocity, which is a sign of CP. When patients were divided into four subgroups, prevalence RD was the lowest in patients with negative cytology and positive CP (p = 0.012).

### Predictors for ACM and RD

Multivariate Cox regression analysis was performed to investigate independent predictors for ACM and RD. Predictors for ACM are shown in Table 2. In multivariate Cox regression analysis, the independent predictors for ACM were male gender (HR; hazard ratio: 1.48, 95% CI; confidential interval: 1.07–2.05, p = 0.019) and positive cytology (HR: 1.86, 95% CI: 1.28–2.70, p = 0.001). In the model using subgroups according to CP and cytology, patients with negative cytology and positive CP were associated with the lowest risk for ACM compared to those with both positive results, even after adjusting for gender and cardiac tamponade (HR: 0.39, 95% CI: 0.20–0.72, p = 0.005).

**Table 2:** Cox regression analysis for predictors of all-cause death.

Predictor	Univariate		Multivariate	
	HR (95% CI)	P-value	HR (95% CI)	P-value
Model 1				
Male gender	1.33 (0.97–1.83)	0.077	1.48 (1.07–2.05)	0.019
Cardiac tamponade	1.48 (0.97–2.25)	0.070	1.26 (0.82–1.94)	0.302
Cytology positive	1.85 (1.30–2.61)	0.001	1.86 (1.28–2.70)	0.001
CP positive	1.24 (0.91–1.70)	0.167	1.07 (0.78–1.46)	0.698
Model 2				
Male gender	1.33 (0.97–1.83)	0.077	1.48 (1.07–2.05)	0.017
Cardiac tamponade	1.48 (0.97–2.25)	0.070	1.36 (0.88–2.11)	0.169
Subgroups according to CP and cytology				
Cytology (+), CP (–)	1.0	-	1.0	-
Cytology (+), CP (+)	1.49 (0.99–2.23)	0.055	1.46 (0.97–2.20)	0.067
Cytology (–), CP (–)	0.83 (0.52–1.34)	0.444	0.85 (0.52–1.39)	0.518
Cytology (–), CP (+)	0.41 (0.21–0.78)	0.007	0.39 (0.20–0.74)	0.005

HR, hazard ratio; CI, confidence interval; CP, constrictive physiology.

Predictors for RD are shown in Table 3. Positive cytology was also an independent predictor for RD in multivariate Cox analysis (p = 0.005). Moreover, the presence of cardiac tamponade and CP after pericardiocentesis were independent predictors for 60% and 66% risk reduction respectively and positive cytology was 3-fold increased risk for RD in the multivariate analysis (p = 0.037, p = 0.012, and p = 0.015, respectively). In the model using subgroups according to CP and cytology, patients with negative cytology and positive CP were associated with the lowest risk for RD compared to those with positive cytology and negative CP, even after adjusting for gender and cardiac tamponade (HR: 0.07, 95% CI: 0.01–0.56, p = 0.012).

**Table 3:** Cox regression analysis for predictors of repeat drainage of pericardial effusion.

Predictor	Univariate		Multivariate	
	HR (95% CI)	P-value	HR (95% CI)	P-value
Model 1				
Male gender	0.68 (0.33–1.41)	0.296	0.78 (0.37–1.65)	0.518
Cardiac tamponade	0.60 (0.27–1.31)	0.196	0.40 (0.17–0.95)	0.037
Cytology positive	1.95 (0.85–4.45)	0.114	3.01 (1.25–7.59)	0.015
CP positive	0.42 (0.19–0.95)	0.038	0.34 (0.15–0.79)	0.012
Model 2				
Male gender	0.68 (0.33–1.41)	0.296	0.75 (0.36–1.58)	0.454
Cardiac tamponade	0.60 (0.27–1.31)	0.196	0.42 (0.18–0.96)	0.041
Subgroups according to CP and cytology				
Cytology (+), CP (–)	1.0	-	1.0	-
Cytology (+), CP (+)	0.37 (0.15–0.90)	0.029	0.36 (0.15–0.87)	0.024
Cytology (–), CP (–)	0.46 (0.18–1.16)	0.098	0.36 (0.13–0.96)	0.040
Cytology (–), CP (+)	0.09 (0.01–0.67)	0.019	0.07 (0.01–0.56)	0.012

HR, hazard ratio; CI, confidence interval; CP, constrictive physiology.

### Comparisons of outcomes according to the results of CP and cytology

**Fig 1:**
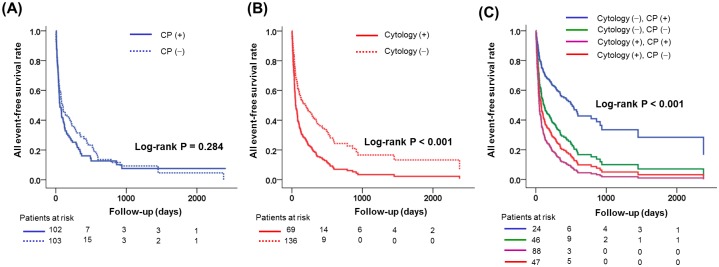
Comparison of all combined events according to the presence of constrictive physiology (A), positive cytology (B) and four groups divided by the results of constrictive physiology and cytology. CP, constrictive physiology.

Fig 1 shows the comparison of all combined events according to the presence of CP and positive cytology in the entire studied malignancy population. There was no significant difference in all combined event-free survival in patients with and without CP (Fig 1A). All combined events were more frequent in patients with positive cytology than in those with negative cytology (Fig 1B). Fig 1C presents the difference in all combined event-free survival among the subgroups according to cytology and CP. Among the four subgroups, patients with negative cytology and positive CP showed the most favorable all event-free survival rates (p < 0.001).

**Fig 2:**
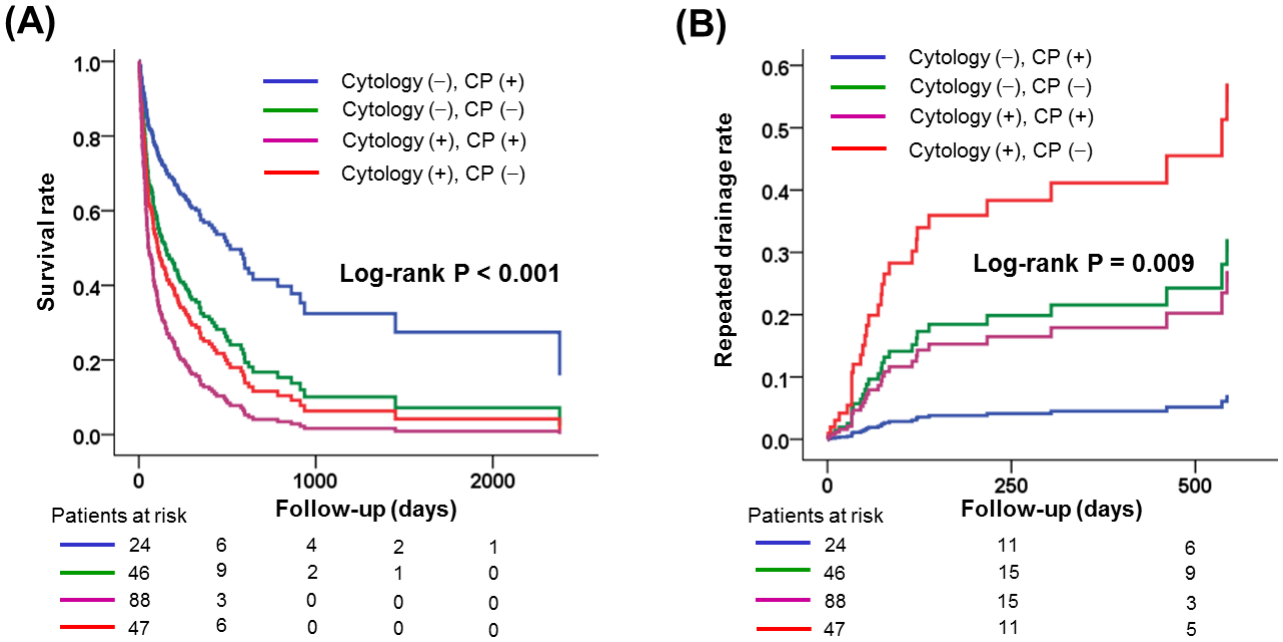
Comparison of survival (A) and repeated drainage for pericardial effusion (B) according to the presence of constrictive physiology and positive cytology. CP, constrictive physiology.

Fig 2 presents ACM and RD according to cytology and CP. Similar to all combined events, patients with negative cytology and positive CP showed the most favorable survival rates and lowest RD rates. In contrast, positive cytology and negative CP showed the most unfavorable survival rates and the highest RD rates among the four subgroups.

**Fig 3:**
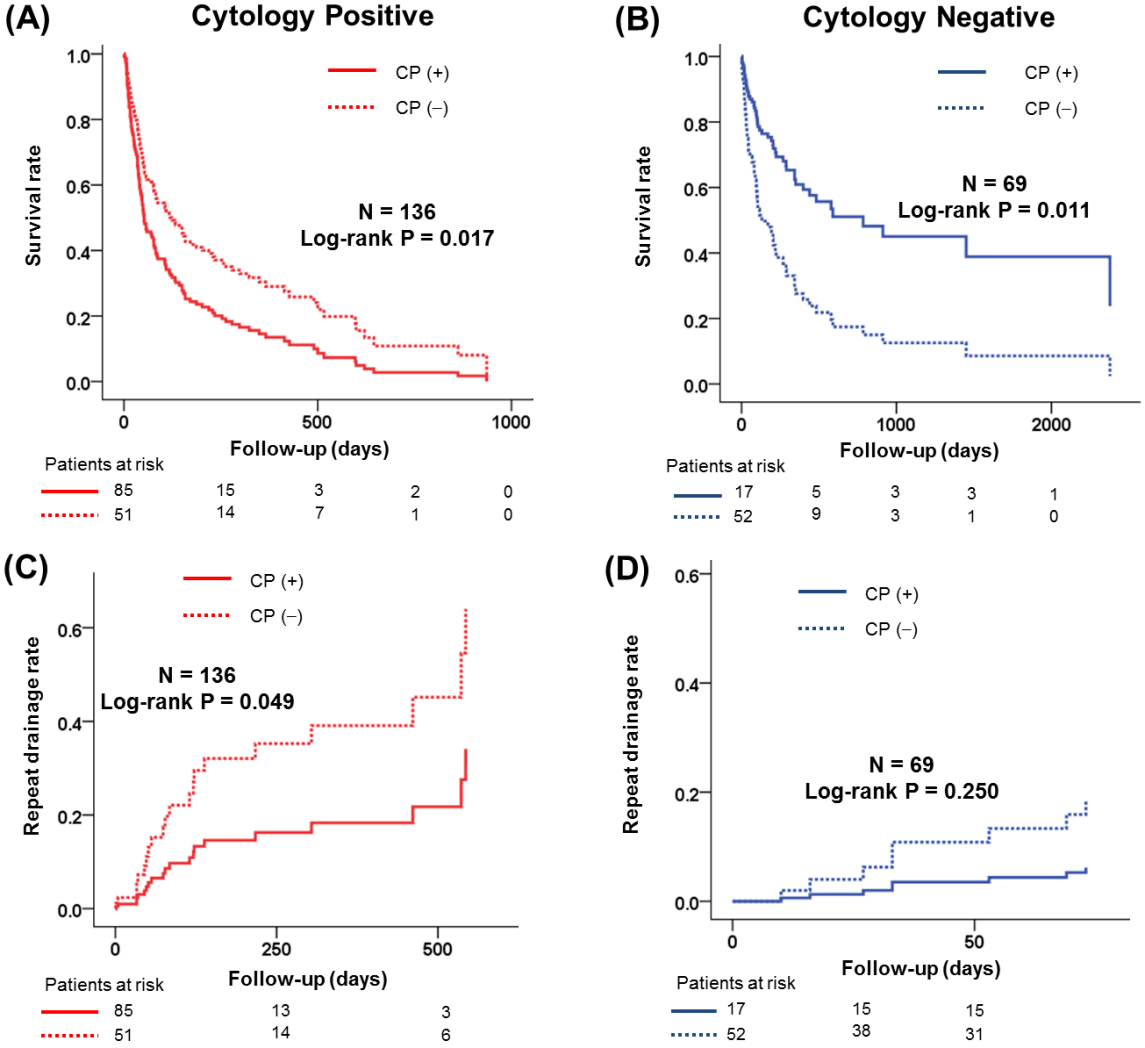
Comparison of survival and repeated drainage for pericardial effusion according to the presence of constrictive physiology. Patients with positive cytology (A, C) and patients with negative cytology (B, D). CP, constrictive physiology.

Since pericardial fluid cytology was an important predictor for all ACM and RD in Cox regression analysis, we divided the population into two categories of positive and negative cytology, and compared ACM and RD. Fig 3 shows a comparison of survival and RD according to the presence of CP in positive cytology (Fig 2A and 2C) and negative cytology (Fig 2B and 2D) group. The survival rate was lower in the CP group compared to the non-CP group in the positive cytology population (p = 0.017). However, in contrast, the survival rate was reversed between CP and non-CP groups among the subgroup of negative cytology (p = 0.010). RD was much higher in the non-CP group in the cytology positive population (p = 0.049), although it was not significantly different among the negative-cytology group according to the presence of CP. Patients with CP in positive cytology also demonstrated a very low rate of RD during follow-up, which was almost similar to that of the cytology-negative group.

### Pericardial thickening and tumor infiltration

CT for the thoracic area including the pericardium was performed for 180 patients among all those included. Table 4 shows comparison of CT and clinical findings in patients with negative or positive cytology according to the presence of CP in the subgroup who underwent chest CT. Pericardial thickening was more frequent in the positive CP group among patients with negative cytology, as well as those with positive cytology (all, p < 0.001). Irregular pericardial nodularity or pericardial tumor infiltration, suggesting malignant effusion, was more prevalent in the CP group only in patients with positive cytology (p = 0.004). In contrast, its prevalence was lower in the CP group among cytology-negative patients, although this was not statistically significant (37% vs. 24%, p = NS).

**Table 4:** Comparison of computed tomography and clinical findings in patients with negative or positive cytology according to the presence of constrictive physiology in the subgroup of 180 patients who underwent chest computed tomography.

Variables	Cytology positive (n = 120)			Cytology negative (n = 60)		
	CP negative (n = 47)	CP positive (n = 73)	P-value	CP negative (n = 43)	CP positive (n = 17)	P-value
Pericardial thickening, n (%)	11 (23)	58 (79)	<0.001	4 (16)	12 (71)	<0.001
Irregular pericardial nodularity or pericardial tumor infiltration, n (%)	30 (64)	63 (86)	0.004	16 (37)	4 (24)	0.311
All-cause mortality, n (%)	37 (79)	70 (96)	0.011	41 (95)	8 (47)	0.012
Repeated drainage, n (%)	14 (30)	7 (10)	0.003	7 (16)	1 (6)	0.669

CP, constrictive physiology.

**Fig 4:**
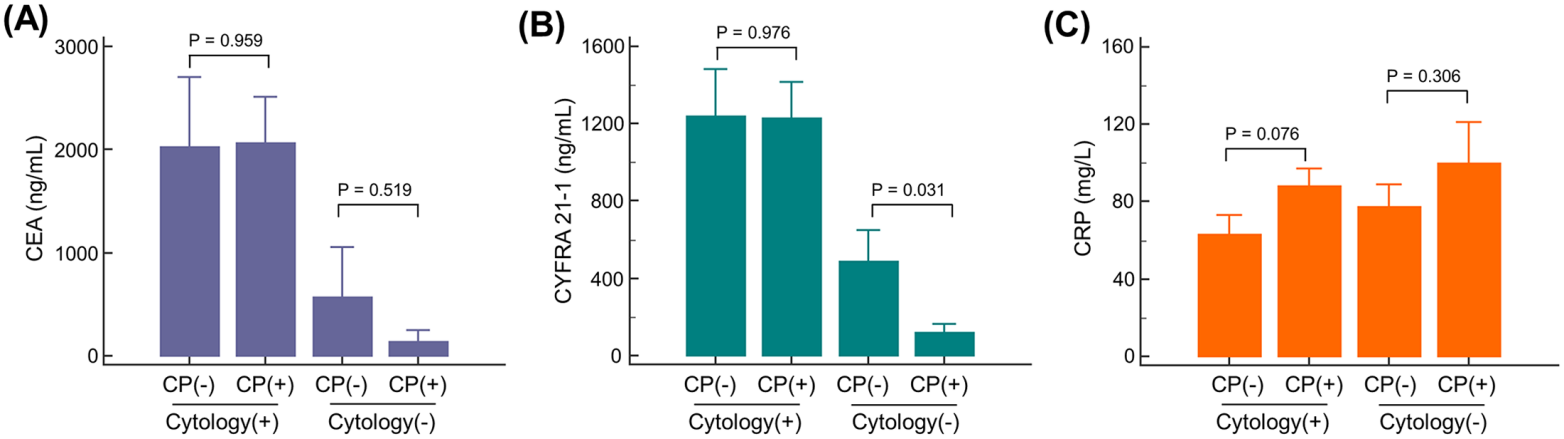
Comparison of pericardial effusion carcinoembryonic antigen (CEA) and cytokeratin fragment 19 (CYFRA 21–1) tumor markers (A, B) and serum C-reactive protein (C) according to the presence of constrictive physiology and cytology. CP, constrictive physiology; CEA, carcinoembryonic antigen; CYFRA 21–1, cytokeratin fragment 19; CRP, C-reactive protein.

Fig 4 presents a comparison of CEA and CYFRA 21–1 in PE and serum CRP according to presence of cytology and CP. CYFRA 21–1 measured in drained PE was significantly higher in patients without CP compared to those with CP in the subgroup of patients with negative cytology (p = 0.031). Serum CRP, a surrogate marker for systemic inflammation, demonstrated higher values in patients with CP in both cytology-positive and cytology-negative groups, although this was not statistically significant. In addition, in the overall study population regardless of cytologic result, CRP was higher in CP groups than in non-CP groups (70.2 ± 66.5 mg/L vs. 93.4 ± 83.0 mg/L, p = 0.038).

## Discussion

The principal finding of the current study is that CP after pericardiocentesis demonstrated differential impacts on ACM and RD in patients with advanced malignancies. The presence of CP in patients with negative cytology conferred the most favorable survival and the lowest rate of RD. In contrast, the presence of CP in patients with positive cytology was associated with poor survival.

### Cancer and PE

Malignant involvement of the pericardium is observed in 2–31% of autopsies of cancer patients,[19] and pericardial disease is a relatively common complication of malignancy that may lead to fatal cardiac tamponade. A large amount of PE is frequently shown in malignancy patients, and one of the leading etiologies of PE requiring pericardiocentesis is malignancy[20]. Meanwhile, PE in malignancy patients does not always guarantee a malignant PE, and the differentiation of malignant PE from nonmalignant PE is not easy, contrary to our expectation. Nonmalignant pericardial disease may be found in as many as 7% of the patients with malignancies at postmortem examination and a significant proportion of patients with pericardial disease and malignancies have a nonmalignant or radiation-related cause of pericardial disease[21].

Currently, the diagnosis of malignant effusion largely depends on cytologic or pathologic examination. Although it has been reported that catheter-based cytology is not inferior to surgical biopsy by pericardiectomy for the diagnosis of malignant PE[22,23], cytologic examination has been shown to be diagnostic of malignancy only in about 85% of patients[21]. Therefore, 15% of malignant effusions can present false-negative results on pericardial fluid cytologic examination.

### CP after pericardiocentesis

CP is an echocardiographic finding suggesting constrictive pericarditis. However, it is not only found in patients with symptomatic constrictive pericarditis but also more commonly observed in rather benign conditions, especially in post-cardiac surgery patients when assessed with a comprehensive echocardiographic examination[9]. CP after pericardiocentesis is a hallmark of effusive-constrictive pericarditis, an uncommon pericardial syndrome in which constriction of the heart by the visceral pericardium occurs in the presence of tense effusion in a free pericardial space[24]. However, it is not clear whether all CPs in the current study resulted from effusive-constrictive pericarditis, as it is a relatively uncommon disease. The prevalence of effusive-constrictive pericarditis has been reported to be 6.9% among patients with clinical tamponade[24] in contrast to our study population, which had a high prevalence of CP after pericardiocentesis of up to 50%. Therefore, in the current study, CP may be associated with various etiologies, such as effusive-constrictive pericarditis, transient pericarditis, and restrictive motion of the pericardium due to direct tumor invasion, all of which are suggested by the various CT findings of this study.

### CP in malignancies and survival

We found that positive cytology was associated with ACM, similar to the previous study[10]. Moreover, positive cytology was also related to RD, as expected. However, interestingly, features of CP after pericardiocentesis showed a differential prognostic impact according to cytology results. In patients with positive cytology, CP was associated with unfavorable survival, in contrast to patients with negative cytology who showed favorable survival outcomes with CP. This differential impact of CP might result from the different pathogeneses that lead to the development of CP in malignant PE and nonmalignant PE.

CP in malignant PE might result from the restricted motion of the pericardium due to infiltration of cancer cells and adhesion of the pericardium to the myocardium with tumor invasion rather than inflammation. Therefore, in positive cytology patients, CP implies broad and wide invasion of the tumor to the pericardium and might be associated with high ACM, as also shown in higher prevalence of irregular pericardial nodularity or pericardial tumor infiltration on CT. On the contrary, nonmalignant pericardial effusions, such as radiation-induced pericarditis, idiopathic pericarditis, drug-induced pericarditis and infection might be associated with pericardial inflammation and resultant CP. The survival of patients with malignancy and nonmalignant PE has been shown to be better than for those with malignant pericarditis[21]. This subgroup of patients which can be identified by negative cytology with positive CP might have favorable survival in the current study, since the etiology of CP is not malignant.

Another distinct group, patients with both negative cytology and CP showed high ACM during follow-up, which is largely comparable to that of cytology-positive patients. We suggest that this subgroup of patients includes patients with false negative cytologic results for malignant cells, and therefore, they showed poor outcomes in ACM. The high prevalence of irregular pericardial nodularity and pericardial tumor infiltration on CT images and the higher values of PE tumor markers in non-CP patients with negative cytology might support our hypothesis. Thus, our data also suggest mixed etiologies of CP in malignancy patients and potential differences between CP and non-CP groups in patients with negative cytology.

### CP and recurrence of PE

Retrospective studies have shown that simple pericardiocentesis in patients with malignant PE have recurrence rates are as high as 90%[2–7], and the recurrence rate was much greater in patients with malignant PE compared with those without malignancy. Therefore, several researchers even recommend surgical pericardiotomy as the definitive treatment for malignant PE. Several studies have been performed to identify the predictors for PE recurrence. The absence of extended catheter drainage, incomplete drainage, loculated effusions and malignancy have been suggested to be predictors for recurrence in PE[3]. The RD of PE was only 14% in the studied population, although all studied patients were diagnosed with advanced malignancies that were usually associated with high recurrence of PE. Tsang et al.[4] reported that the recurrence rate of PE or persistent drainage that necessitated secondary management was low, down to 11.5% when pericardiocentesis was performed with extended catheter drainage. As the same method was performed in our study population as well, extended catheter drainage for managing PE might be crucial to avoid recurrent PE in patients with advanced malignancy.

In the current study, we found that the presence of CP predicted a very low rate of RD for both positive and negative cytology. The reason for this phenomenon is not clear. One possible explanation may be that the long-term survival rate was poorest in the positive-cytology group; therefore, the chances of RD were low due to the limited life expectancy of the population with malignant PE. Therefore, the wisest strategy appears to be simply monitoring the cases clinically and, if the large PE recurs, draining the PE by employing simple pericardiocentesis alone rather than by aggressive interventions such as balloon pericardiotomy or surgical drainage in populations with both positive results, which suggest advanced cancer with pericardial invasion and thereby indicate a low rate of long-term survival.

Another possible explanation for the low incidence of RD is that the etiology of PE in patients with CP may be different from those without CP, which is associated with transient inflammation of the pericardium and may be transient itself. This theory can be supported by the CT findings of the patients with negative cytology and positive CP, which showed the lowest prevalence of irregular pericardial nodularity or pericardial tumor infiltration and the lowest prevalence of RD. In addition, the fact that CRP level was higher in the CP group than in the non-CP group regardless of cytologic result also advocates the theory that CP might imply an inflammatory process that would be associated with a low rate of RD. This phenomenon also explains the lower RD rate in patients with both positive cytology and CP compared to those with positive cytology and negative CP. In fact, as expected, patients with negative cytology and positive CP demonstrated the lowest PE tumor marker values and the highest CRP levels. Therefore, we also suggest that CP in negative cytology might be associated with inflammation rather than malignant effusion. Further studies investigating inflammatory markers in PE and applying anti-inflammatory medications for those patients would be helpful in identifying the role of inflammation of the pericardium and effusion in the recurrence of large PE in patients with malignancies.

### Limitations

The main limitation of the current study is that this is a retrospective study. Pericardial effusion in cancer patients is frequently related to various clinical conditions, such as radiation, inflammation, opportunistic infections, drugs, hypoalbuminemia, and involvement of the lymphatic system. However, due to retrospective nature of the current study and the complexity of the study population (specifically, variance in chemotherapy regimens and general conditions), it was difficult to describe our observations in a consistent manner. The choice of imaging, procedural technique, and the duration of pericardial catheter drainage were all operator dependent. The types of cancer were varied, although lung cancer was the most common. This study was performed in malignancy patients requiring pericardiocentesis, and the result of this study cannot be directly applied to other population without malignancies or small amounts of pericardial effusion. Clinical findings suggesting symptomatic constrictive pericarditis were lacking due to limited medical records associated with this retrospective analysis covering a long period.

## Conclusions

CP after pericardiocentesis is common, but does not always imply poor survival or the need for RD in patients with malignancies. On the contrary, CP in patients with negative cytology showed favorable survival and low rate of RD for PE. Comprehensive echocardiographic evaluation for CP after pericardiocentesis would be helpful for predicting prognosis in patients with advanced malignancies.
